# 111 Use of Neck Tissue Expansion for Post-burn Reconstruction

**DOI:** 10.1093/jbcr/iraf019.111

**Published:** 2025-04-01

**Authors:** Tina Moon, Matthew DePamphilis, Thomas Guzman, Evangeline Adjei-Danquah, Daniel Driscoll

**Affiliations:** Massachusetts General Hospital; Shriners Children’s - Boston; Shriners Children’s - Boston; Shriners Children’s - Boston; Shriners Children’s - Boston

## Abstract

**Introduction:**

Neck tissue expansion is a versatile tool for aesthetic and functional post-burn reconstruction when addressing burn scars in the neck or lower two-thirds of the face. Tissue expansion offers advantages over other reconstructive modalities such as the opportunity to resurface scars with excellent color and texture match of expanded skin while limiting donor site morbidity. The aim of this study was to evaluate our 20-year institutional experience with neck tissue expansion for postburn reconstruction.

**Methods:**

A retrospective review was conducted on consecutive patients aged 0–21 years old admitted to a specialized pediatric burn hospital from January 2004 to January 2024 for postburn reconstruction with neck tissue expansion. Complications were categorized as major or minor, with major complications defined as those which required premature removal of the tissue expander.

**Results:**

A total of 31 neck tissue expanders were placed in 19 patients. The mean age at reconstruction was 15 years (range 4 to 21 years), 53% were female, and 47% were international referrals. The average total body surface area (TBSA) was 30% (range 7% - 66%). Sixty three percent of patients had sustained a flame burn mechanism of injury. The time period from burn injury to reconstruction averaged 6 years and the large majority (81%) had undergone prior reconstructive efforts. The average area to be reconstructed was 114 cm2 and patients went through expansion processes for an average of 102 days. The major complications rate was 0%. The minor complication rate was 26%, which included four cases of delayed wound healing, two seromas, one implant exposure, and one infection. These all resolved with operative intervention and resulted in successful reconstruction. Patients were followed postoperatively for an average of 4 years.

**Conclusions:**

Burn scar contracture in the neck can lead to significant functional and aesthetic deformities. Our results demonstrate how tissue expansion of the neck can be a successful and versatile treatment modality for postburn reconstruction.

**Applicability of Research to Practice:**

Reconstruction of neck burn scar contracture using tissue expansion is a safe modality that provides similar tissue while limiting donor site morbidity.

**Funding for the Study:**

N/A

